# Use of wearable sensors to assess compliance of asthmatic children in response to lockdown measures for the COVID-19 epidemic

**DOI:** 10.1038/s41598-021-85358-4

**Published:** 2021-03-15

**Authors:** Panayiotis Kouis, Antonis Michanikou, Pinelopi Anagnostopoulou, Emmanouil Galanakis, Eleni Michaelidou, Helen Dimitriou, Andreas M. Matthaiou, Paraskevi Kinni, Souzana Achilleos, Harris Zacharatos, Stefania I. Papatheodorou, Petros Koutrakis, Georgios K. Nikolopoulos, Panayiotis K. Yiallouros

**Affiliations:** 1 Respiratory Physiology Laboratory, Medical School, Shacolas Educational Center of Clinical Medicine, University of Cyprus, Palaios Dromos Lefkosias-Lemesou 215/6, 2029 Aglantzia, Nicosia, Cyprus; 2 Institute of Anatomy, University of Bern, Bern, Switzerland; 3 Medical School, University of Crete, Heraklion, Crete Greece; 4 Cyprus International Institute for Environmental and Public Health, Cyprus University of Technology, Limassol, Cyprus; 5 Cellock LTD, Nicosia, Cyprus; 6 Embrace Tech LTD, Nicosia, Cyprus; 7 Department of Epidemiology, Harvard T.H. Chan School of Public Health, Harvard University, Boston, MA USA; 8 Department of Environmental Health, Harvard TH Chan School of Public Health, Harvard University, Boston, USA; 9 Medical School, University of Cyprus, Nicosia, Cyprus

**Keywords:** Biomedical engineering, Public health, Environmental impact

## Abstract

Between March and April 2020, Cyprus and Greece health authorities enforced three escalated levels of public health interventions to control the COVID-19 pandemic. We quantified compliance of 108 asthmatic schoolchildren (53 from Cyprus, 55 from Greece, mean age 9.7 years) from both countries to intervention levels, using wearable sensors to continuously track personal location and physical activity. Changes in ‘fraction time spent at home’ and ‘total steps/day’ were assessed with a mixed-effects model adjusting for confounders. We observed significant mean increases in ‘fraction time spent at home’ in Cyprus and Greece, during each intervention level by 41.4% and 14.3% (level 1), 48.7% and 23.1% (level 2) and 45.2% and 32.0% (level 3), respectively. Physical activity in Cyprus and Greece demonstrated significant mean decreases by − 2,531 and − 1,191 (level 1), − 3,638 and − 2,337 (level 2) and − 3,644 and − 1,961 (level 3) total steps/day, respectively. Significant independent effects of weekends and age were found on ‘fraction time spent at home’. Similarly, weekends, age, humidity and gender had an independent effect on physical activity. We suggest that wearable technology provides objective, continuous, real-time location and activity data making possible to inform in a timely manner public health officials on compliance to various tiers of public health interventions during a pandemic.

## Introduction

Following several coronavirus outbreaks during the last years^1^, a novel coronavirus named SARS-CoV-2 presented in Wuhan, China in December 2019, causing severe disease (COVID-19) with high fatality rates, especially, amongst the elderly and people with comorbidities^2^. The virus rapidly spread all over the world and on the 11th of March 2020, WHO characterized COVID-19 outbreak as a pandemic^3^.

In the absence of an effective vaccine or specific antiviral drugs against COVID-19^4^, it seems that the only strategy to control the pandemic are public health interventions implemented at the community or national level. The interventions may range from simple isolation of disease carriers, quarantine of contacts and hand hygiene measures to ban of mass gatherings, social distancing and finally to complete lockdown and community quarantine (cordon sanitaire)^5–7^. These measures coupled with extraordinary travel restrictions from national governments are often in disaccord with international and human rights law^8^. During the first peak of the pandemic, affected countries chose different levels of interventions based, among others, on national risk assessments of estimated number of patients and capacity for hospitalization and critical care support^9,10^. The evolution of the pandemic over the last months demonstrated that timely interventions were effective to delay the spread of COVID-19^11,12^, as it was also shown in previous flu and SARS epidemics^13–15^.

The compliance of the population to non-pharmaceutical interventions plays a catalytic role in the successful containment of the virus^16^. However, it is difficult to monitor and understand population’s compliance to behavioural changes forced by interventions during an emerging pandemic^17–19^. To date, several parameters have been used as indirect indicators of societal behaviour and compliance to public health measures, such as the reduction of outdoor pollution^20,21^, decrease in traffic accidents^22^ or even quietening of human activity signals as measured by seismometers worldwide^23^. In the context of complete lockdown measures, where citizens are advised to reduce their mobility and stay at home, wearable sensors measuring physical activity levels and time spent at home may serve as a direct indicator for citizens’ compliance to the measures.

Patients with chronic respiratory disorders, like asthma, are considered to be at increased risk for severe COVID-19 disease^24,25^ and from the beginning of the pandemic they were advised to meticulously comply to restriction measures. In the pediatric population, asthma is the most common chronic disorder and although COVID-19 is milder in children, the burden of the disease on public health may be significant^26^. Furthermore, it is known that children may have more difficulties to comply to restriction measures^27^*,* with their compliance depending substantially on the whole family’s attitude towards the measures. The aim of our study was to quantify mobility changes in response to COVID-19 lockdown measures of schoolchildren with asthma in Cyprus and Greece, by continuously tracking their location and activity, using wearable sensors.

## Results

### Participants’ characteristics

For 2020 study period, a total of 108 (57% males) asthmatic children, 53 in Cyprus and 55 in Greece, with an average age of 9.2 years were enrolled in the study, and contributed data between February 3rd and April 26th, 2020. All children had a physician’s diagnosis of asthma, while 53% also reported wheezing episode(s) during the past 12 months, 45% unscheduled medical visit(s) for asthma, 26% emergency room visits for asthma, and 20% daily preventive anti-asthma medication during the past 12 months. Among study participants, 43% were characterized as having asthma severity 1, 43% asthma severity 2 and 15% asthma severity 3 (Table 1). For the 2019 study period, from February 3rd to April 26th, we measured mobility for 39 and 52 asthmatic children (59% males) in Cyprus and Greece, respectively, with an average age of 9.3 years.

**Table 1:** Basic demographic and clinical characteristics of asthmatic children in Cyprus and Greece included in the study for the 2020 study period.

Parameter	Asthmatic children (Cyprus, n = 53)	Asthmatic children (Greece, n = 55)
**Demographic**		
M (%)	35 (66%)	27 (49%)
Age, years*	9.3 (1.7)	9.11 (1.8)
Weight, kg*	39.3 (19.0)	35.9 (9.6)
Height, cm*	137.2 (21)	134.8 (10.2)
BMI, kg/m^2^*	20.3 (8.3)	19.5 (3.3)
**Asthma eligibility criteria**		
Physician diagnosis of Asthma	53 (100%)	55 (100%)
Wheezing episodes	35 (66%)	22 (40%)
Daily preventive medication	10 (19%)	12 (22%)
Unscheduled physician visits for Asthma	34 (64%)	15 (27%)
ER^†^ visits for Asthma	10 (19%)	18 (33%)
**Asthma severity status‡**		
Asthma severity 1	25 (47%)	21 (38%)
Asthma severity 2	20 (38%)	26 (47%)
Asthma severity 3	8 (15%)	8 (15%)

*Values are presented as Mean (Standard Deviation). ^†^ER: Emergency Department.

^‡^Asthma Severity 1: Physician diagnosis plus one other eligibility criterion, Asthma Severity 2: Physician diagnosis plus two other eligibility criteria, Asthma Severity 3: Physician diagnosis plus three or more other eligibility criteria.

### Unadjusted analysis

**Figure 1:**
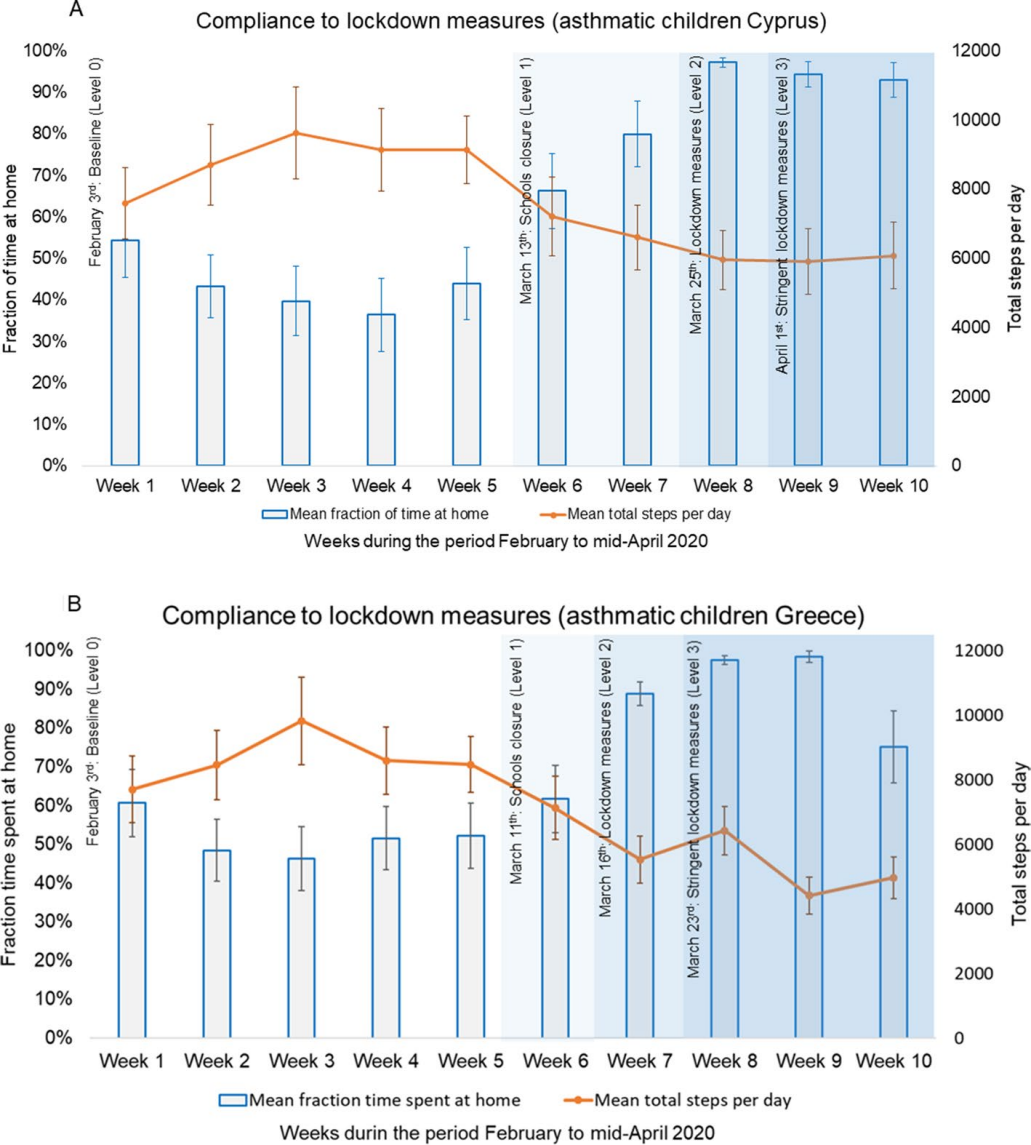
Changes in mobility in response to public health interventions among asthmatic children. Weekly averages of fraction time spent at home and steps/day, before and during three levels of public health interventions in asthmatic children in Cyprus (**A**) and Greece (**B**).

**Figure 2:**
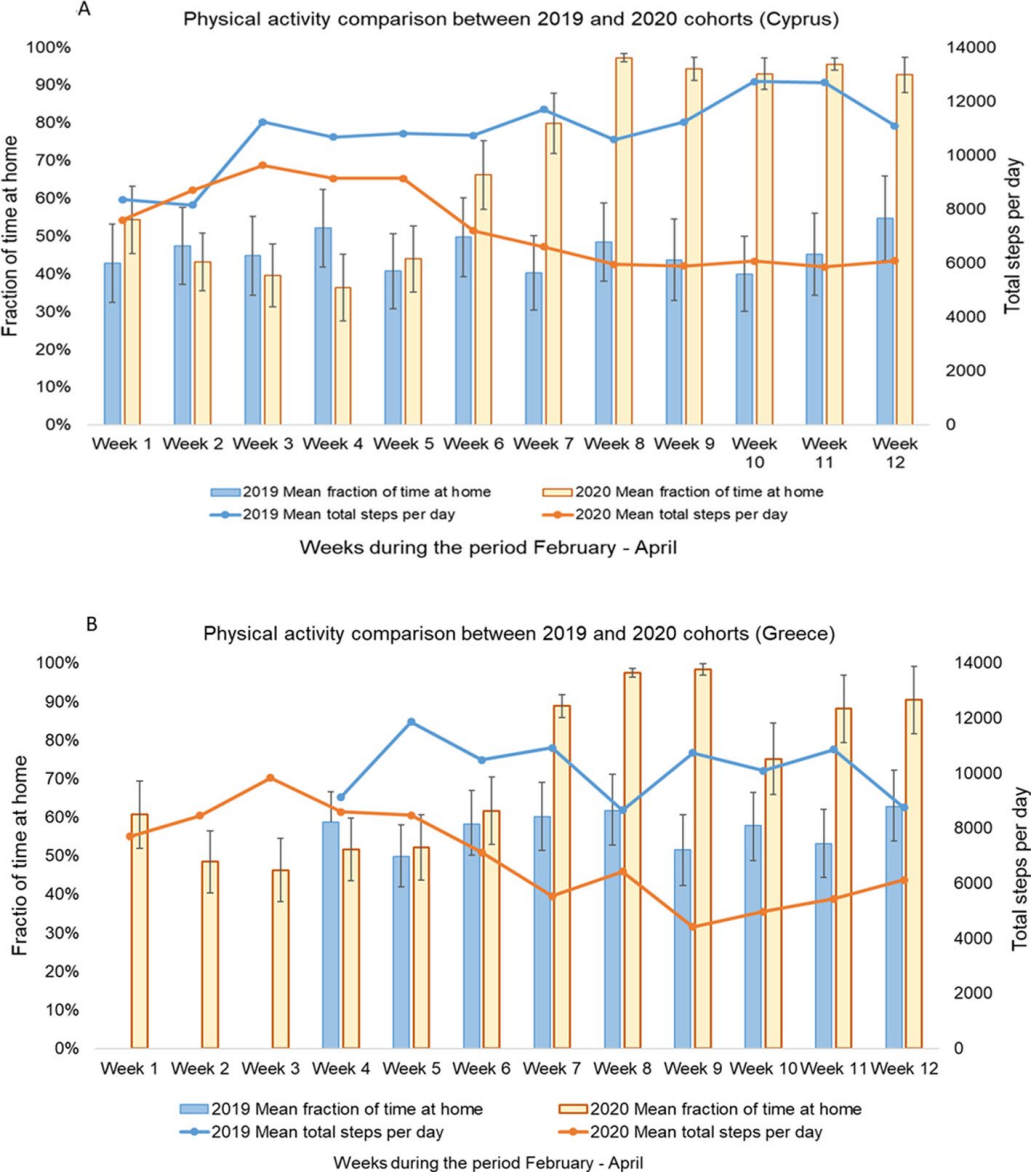
Comparison of mobility of asthmatic children recorded in February–April 2019 and February–April 2020 in Cyprus and Crete. Weekly averages of fraction time spent at home and steps/day during the same period of 2019 and 2020 in Cyprus (**A**) and Greece (**B**) (February–April).

Overall, in both countries there were significant changes in the observed fraction time spent at home and total steps/day with introduction of public health intervention levels for the study year 2020. The observed mean fraction time spent at home among asthmatic children in Cyprus and Greece during baseline period was 43.8% (95%CI: 40.5–47.1%) and 52.4% (95%CI: 49.4–55.4%) respectively. In Cyprus, the observed fraction time spent at home significantly increased to 88.9% (95%CI: 85.7–92.1%) during level 1, to 95.5% (95%CI: 93.8–97.2%) during level 2 and 94.1% (95%CI: 92.5–95.7%) during level 3 of interventions. In Greece, introduction of level 1 public health interventions was characterized by an increase in the observed fraction time spent at home to 71.4% (95%CI: 60.4–82.5%), while during level 2 and 3 interventions the fraction time spent at home increased further to 84.9% (95%CI: 80.3–89.4%) and 89.6% (95%CI: 87.0–92.3%) respectively (Table 2, Fig. 1). In Cyprus, the observed total steps/day reduced significantly with introduction of each level of public health interventions from 8,996 (95%CI: 8,567–9,425) at baseline, to 6,499 (95%CI: 5,832–7,166) at level 1, 6,248 (95%CI: 5,683–6,812) at level 2 and 6,270 (95%CI: 5,814–6,727) at level 3. Similarly, in Greece, the observed total steps/day reduced with each level of public health interventions from 8,527 (95%CI: 8,145–8,908) at baseline, to 6,864 (95%CI: 5,689–8,040) at level 1, 5,533 (95%CI: 4,769–6,297) at level 2 and 5,439 (95%CI: 5,051–5,829) at level 3 (Table 2, Fig. 1). The same trend was also observed for fraction time spent at home and total steps per day in both countries when the change across levels of intervention were calculated separately for each category of asthma severity. Data are presented in detail in Supplementary Table 1. This pattern was in sharp contrast to the normal mobility pattern of the asthmatic children cohorts in Cyprus and Greece during the same study period for the study year 2019, where the fraction time spent at home and total steps per day were quite stable throughout the same weeks of the year (Fig. 2).

**Table 2:** Observed fraction time spent at home and total steps per day across levels of intervention for Covid-19 in Cyprus and Greece. Values are presented as mean (95% Confidence Interval).

Intervention	Parameter	Statistical significance		Parameter	Statistical significance	
	Fraction time spent at home	Compared to baseline	Compared to previous level	Steps per day	Compared to baseline	Compared to previous level
**Asthmatic children (Cyprus) (n = 53)**						
Baseline (Level 0)	43.8% (40.5%; 47.1%)	–	–	8996 (8567; 9425)	–	–
Level 1	88.9% (85.7%; 92.1%)	< 0.001	< 0.001	6499 (5832; 7166)	< 0.001	< 0.001
Level 2	95.5% (93.8%; 97.2%)	< 0.001	0.151	6248 (5683; 6812)	< 0.001	0.999
Level 3	94.1% (92.5%; 95.7%)	< 0.001	0.999	6270 (5814; 6727)	< 0.001	0.999

	Fraction time spent at home			Steps per day		
**Asthmatic children (Greece) (n = 55)**						
Baseline (Level 0)	52.4% (49.4%; 55.4%)	–	–	8527 (8145; 8908)	–	–
Level 1	71.4% (60.4%; 82.5%)	0.003	0.003	6864 (5689; 8040)	0.060	0.060
Level 2	84.9% (80.3%; 89.4%)	< 0.001	0.357	5533 (4769; 6297)	< 0.001	0.613
Level 3	89.6% (87.0%; 92.3%)	< 0.001	0.999	5439 (5051; 5829)	< 0.001	0.999

### Adjusted analysis

Based on the mixed effects model, after controlling for several confounders, the adjusted mean increase in time-fractions spent at home in Cyprus were: 41.4% (95%CI: 34.5–48.2%, *p*_value_ < 0.001 compared to baseline) during level 1, 48.7% (95%CI: 42.0–55.5%, *p*_value_ < 0.001 compared to baseline) during level 2, and 45.2% (95%CI: 39.3–51.2%, *p*_value_ < 0.001 compared to baseline) during level 3 of interventions (Table 3). There was no significant increase between level 2 and 3 periods (*p*_value_ = 0.298), while the increase between level 1 and 2 periods was almost significant (*p*_value_ = 0.055). In Greece, the adjusted mean changes in fraction time spent at home were more gradual and moderate, increasing by 14.3% (95%CI: 2.2–26.3%, *p*_value_ = 0.02 compared to baseline) during level 1, 23.1% (95%CI: 11.2–34.9%, *p*_value_ < 0.001 compared to baseline) during level 2, and 32.0% (95%CI: 24.8–39.3%, *p*_value_ < 0.001 compared to baseline) during level 3 interventions (Table 4). The changes between levels 1 and 2 as well as levels 2 and 3 were not statistically significant.

**Table 3:** Fraction time spent at home and total steps per day in response to interventions for Covid-19 among asthmatic children in Cyprus.

Parameter	Fraction time spent at home		Total Steps per day	
	*β* coefficient (95% CI)	Compared to baseline	*β* coefficient (95% CI)	Compared to baseline
Baseline (Level 0) (intercept)	47.1% (18.2%; 76.0%)	–	13,520 (9123–17,917)	–
Level 1	41.4% (34.5%; 48.2%)	< 0.001*	− 2531 (− 3364; − 1698)	< 0.001*
Level 2	48.7% (42.0%; 55.5%)	< 0.001^†^	− 3638 (− 4521; − 2755)	< 0.001^‡^
Level 3	45.2% (39.3%; 51.2%)	< 0.001^§^	− 3644 (− 4428; − 2859)	< 0.001^§^
Gender (male)	1.7% (− 3.2%; 6.5%)	0.502	1024 (53;1944)	0.039
Age (per year increase)	1.4% (0.2%; 2.6%)	0.019	− 378 (− 615; − 143)	0.002
Year (2020)	− 0.6% (− 6.4%; 5.1%)	0.826	− 1560 (− 2796; − 324)	0.013
Weekend	10.9% (8.0%; 13.8%)	< 0.001	− 1002 (1374; − 630)	< 0.001
Temperature (per degree C^0^ increase)	− 0.03% (− 0.07%; 0.15%)	0.909	58.0 (− 6; 122)	0.075
Humidity (per % increase	0.04% (− 0.07%; 0.15%)	0.539	− 29 (− 42; − 15)	< 0.001

Interaction term	*β* coefficient (95% CI)	Compared to baseline	*β* coefficient (95% CI)	Compared to baseline
Weekend # Level 1	− 22.8% (− 33.8%; − 11.8%)	< 0.001	1870 (539; 3202)	0.006
Weekend # Level 2	− 13.0% (− 25.9%; − 0.1%)	0.047	1196 (− 535; 2927)	0.176
Weekend # Level 3	− 18.6% (− 27.4%; − 9.8%)	< 0.001	1361 (191; 2531)	< 0.001

*p < 0.001, compared to previous level. ^†^*p* = 0.055, compared to the previous level. ^‡^*p* = 0.021, compared to the previous level, ^§^*p* > 0.05, compared to the previous level.

**Table 4:** Fraction time spent at home and total steps per day in response to interventions for Covid-19 among asthmatic children in Greece.

Parameter	Fraction time spent at home		Total Steps per day	
	*β* coefficient (95% CI)	Compared to baseline	*β* coefficient (95% CI)	Compared to baseline
Baseline (Level 0) (intercept)	74.6% (52.3%; 97.0%)	–	13,342 (9182; 17,501)	–
Level 1	14.3% (2.2%; 26.3%)	0.020*	− 1191 (− 2641; − 259)	0.108^†^
Level 2	23.1% (11.2%; 34.9%)	< 0.001^†^	− 2337 (− 3679; − 995)	0.001^†^
Level 3	32.0% (24.8%; − 39.3%)	< 0.001^†^	− 1961 (− 2933; − 990)	< 0.001^†^
Gender (male)	2.2% (− 3.4%; 7.7%)	0.442	1064 (− 63; 2191)	0.064
Age (per year increase)	− 1.0% (− 2.6%; 0.5%)	0.191	57 (− 257; 370)	0.722
Year (2020)	− 0.7% (− 7.9%; 6.6%)	0.859	− 2791 (− 4090; − 1492)	< 0.001
Weekend	8.32% (5.2%; 11.4%)	< 0.001	− 1212 (1615; − 809)	< 0.001
Temperature (per degree C^0^ increase)	− 0.6% (− 1.3%; 0.0%)	0.059	21 (− 64; 107)	0.628
Humidity (per % increase)	− 0.1% (− 0.2%; 0.0%)	0.061	− 2 (− 16; 12)	0.763

Interaction term	*β* coefficient (95% CI)	Compared to baseline	*β* coefficient (95% CI)	Compared to baseline
Weekend # Level 1	− 12.7% (− 37.2%; − 11.8%)	0.310	149 (− 2739; 3037)	0.006
Weekend # Level 2	− 9.5% (− 32.5%; 13.5%)	0.416	3704 (1115; 6292)	0.005
Weekend # Level 3	− 9.6% (− 19.8%; 0.5%)	0.063	1433 (96; 2772)	0.036

*p = 0.020, compared to previous level. ^†^*p* > 0.05, compared to the previous level.

In Cyprus, physical activity, expressed in total steps per day, demonstrated an adjusted mean decrease of − 2,531 (95%CI: − 3,364; − 1,698, *p*_value_ < 0.001 compared to baseline) during level 1, − 3,638 (95%CI: − 4,521; − 2,755, *p*_value_ < 0.001 compared to baseline) during level 2, and − 3,644 (95%CI: − 4,428; − 2,859, *p*_value_ < 0.001 compared to baseline) during level 3 (Table 3). The decreases in total steps per day for levels 1 and 2 were statistically significant (*p*_value_ = 0.019), but not for levels 2 and 3 (*p*_value_ = 0.954). In Greece, the adjusted mean decrease of physical activity during level 1 period was − 1,191 (95%CI: − 2,641; − 259, *p*_value_ = 0.108 compared to baseline) steps per day. During level 2 and 3 periods the corresponding decreases were − 2,337 (95%CI: − 3,679; − 995, *p*_value_ = 0.001 compared to baseline) and − 1,961 (95%CI: − 2,933; − 990, *p*_value_ < 0.001 compared to baseline) steps/day, respectively (Table 4). The differences in total steps decreases per day between levels 1 and 2 (*p*_value_ = 0.212) and levels 2 and 3 (p_value_ = 0.576) did not reach statistical significance.

During the baseline period, in the cohort of asthmatic children in Cyprus, we found that fraction time spent at home was significantly higher during weekends as compared to weekdays (mean increase compared to weekdays: 10.9%. 95%CI: 8.0; 13.8%) and with every year of increasing age (mean increase: 1.4%. 95%CI: 0.2; 2.6%). Furthermore, total steps per day were significantly lower during weekends (mean decrease compared to weekdays: − 1,002. 95%CI: − 1,374; − 630), with increasing age (mean decrease: − 378. 95% CI: − 615; − 143), increasing humidity (mean decrease: − 29. 95%CI: − 42; − 15) and in year 2020 as compared to year 2019 (mean decrease: − 1,560. 95%CI: − 2,796; − 324). Finally, total steps per day were significantly higher in males as compared to females (mean increase: 1,024. 95%CI: 53; 1,944) (Table 3). In asthmatic children in Greece, the fraction of time spent at home was also found to be significantly higher during weekends as compared to weekdays (mean increase: 8.32%. 95%CI: 5.2; 11.4%), while steps per day were significantly lower during weekends (mean decrease: − 1,212. 95%CI: − 1,615; − 809) and in year 2020 as compared to year 2019 (mean decrease: − 2,791. 95%CI: − 4,090; − 1,492) (Table 4). In asthmatic children in Cyprus, we found a significant interaction effect of weekends across all levels of interventions on both the fraction time spent indoors and total steps per day. The effect of weekends on fraction time spent indoors changed, significantly, from positive during level 0 (mean increase: 10.9%) to negative during level 1 (mean decrease: − 22.8%, *p*_value_: < 0.001), level 2 (mean decrease: − 13.0%, *p*_value_: 0.047) and level 3 (mean decrease: − 18.6%, *p*_value_: < 0.001). A similar interaction effect was observed on the effect of weekends on total steps per day which changed from negative during level 0 (mean decrease: − 1002) to positive during level 1 (mean increase: 1863, *p*_value_: 0.006), level 2 (mean increase: 1180, *p*_value_: 0.181) and level 3 (mean increase: 1359, *p*_value_: < 0.023), (Table 3). In asthmatic children in Greece we found a significant interaction effect for weekends only in regards to total steps per day. During the baseline period, weekends were associated with a mean decrease of − 1212 steps per day while this effect was reversed in level 1 (mean increase: 149, *p*_value_: 0.919), level 2 (mean increase: 3704, *p*_value_: 0.005) and level 3 (mean increase: 1434, *p*_value_: 0.036), (Table 4).

## Discussion

In this study, we have assessed changes in mobility of asthmatic children in Cyprus and Greece in response to different levels of public health interventions for the COVID-19 pandemic using GPS tracking, pedometer and heart rate sensors embedded in wearable watches. Our data imply that asthmatic children in both countries were highly compliant to public health measures, by changing their everyday routine and limiting their activity and mobility outside their homes.

In asthmatic children in Cyprus, we recorded a steep increase in the fraction of time spent at home from 44% to the very high 95% and a steep decrease in total steps per day from 8,996 to 6,270, demonstrating high compliance to the implemented three levels of interventions. In asthmatic children in Greece, we observed a more gradual, stepwise increase in fraction time spent at home from 52% at baseline to the also high 90% and a similar pattern of gradual decrease of physical activity from 8,527 to 5,439 steps/day. The subtle differences in the findings between the two countries can be explained by differences in the actual measures implemented in each country rather than by real differences in the compliance of asthmatic children to the interventions. In fact, intervention measures in Greece were slightly different to those in Cyprus. In Greece level 2 measures included only ban of public gatherings and closure of shops and worship places, and escalated to include personal transport and movement restrictions only in level 3 period. In Cyprus, personal mobility restrictions were implemented from level 2 measures and became stricter (only one movement per day) in level 3, although the corresponding changes in fraction time spent at home and steps per day between level 2 and level 3 periods were not significant in our study group.

The successful management of the pandemic spread in Cyprus and Greece is probably due to the early introduction of lockdown measures in both countries by March 25 and 23, 2020 respectively, and the high compliance of vulnerable groups, and possibly the general population, who spent the majority of their time at home. The effectiveness of lockdown measures to reduce transmission of COVID-19 in several European countries including Greece was previously reported^28^. A recent modelling study evaluated the impact of the sequence of restrictions posed to mobility and human-to-human interactions on the virus transmission in Italy and found that they have reduced transmission by 45%^29^. As of 26 April 2020, 1.43 and 1.22 deaths per 100,000 population were reported for Cyprus and Greece respectively, which places them among the countries with the lowest mortality rates for COVID-19 in the EU/EEA and UK^30^. Our findings may also relate to those of several recent studies that reported reduced asthma morbidity in children during the COVID-19 pandemic, attributed to the reduction of the overall viral infections because of significant changes in their daily activity^31–34^.

It is well documented that wearable technology is a reliable, objective tool for monitoring numerous diseases and estimating adherence to medication^35^. However, to the best of our knowledge, there are no previous studies examining compliance to public health interventions using wearable devices. Previous reports on adherence to public health interventions during epidemics were based on telephone or mailed interviews and questionnaires^17–19,36^. These conventional tools have inherent limitations such as non-response, recall biases, lack of validation of self-reports, influence by concerns about being recognized as breaching quarantine and low-level spatio-temporal information. In contrast, the use of wearable devices provides objective, continuous, real-time location and activity data making possible to timely inform public health officials on the results of various tiers of public health interventions and ensure adequate decision making in escalating or de-escalating interventions.

Using a mixed effects model, we were able to find independent quantitative effects of several factors on the time participants spent at home (weekends, increasing age) and their physical activity (weekends, increasing age, humidity and gender). We also found an interaction effect of weekends on higher fraction of time spent at home and lower physical activity, which was reversed during the implementation of intervention measures in both countries. One possible explanation is that, under normal conditions, families spend more time out of home on weekdays and more time at home during weekends. During the pandemic period this was reversed, as working parents and children had to work or study at home during weekdays and spend more time for outdoor activities on weekends. However, the change of participants’ daily behavior in both countries and the increase in their mobility on weekends during the enforcement of the lockdown measures requires further investigation. The independent effect of age on increasing fraction time spent at home and lower physical activity and female gender on lower physical activity in asthmatic children in Cyprus, agree with previous studies that reported lower physical activity in asthmatic girls^37^ and older asthmatic children^38^. Year 2020 has been associated with lower physical activity in asthmatic children in both countries, which may relate to a systematic effect of either the recording devices used or implication of environmental factors we have not accounted for in the analysis.

Digital technology has been widely used in the COVID-19 management, including tracking positive cases, informing citizens about the current epidemiological status in their region, performing virtual clinics and diagnostics through telemedicine and in disease modelling using big data analysis^39,40^. Our study presents another promising application of digital technology in the fight against the pandemic, by employing wearable sensors to provide objective and real-time information about population compliance to public health measures. Therefore, in a public health emergency, it is possible to employ wearable technology in a greater sample of the general population and enable rapid measurements of the public reaction to a particular set of interventions. GPS and physical activity data for example, are collected anonymously by telephone companies from their smartphone customers and theoretically may provide information about population’s attitude and adherence to interventions taken in pandemics and/or natural disasters. Towards this aim, in Israel a cell-phone tracking system was used in SARS-CoV-2 positive individuals to facilitate contacts tracing^41^, while data from smartphone users in Italy have been analysed to estimate citizen mobility during the lockdown^42^. In this direction, future applications of wearable digital technology may include the development of appropriate tools and infrastructure for efficient, systematic integration and interoperability of different health and behaviour data sources into existing public health systems. This approach will facilitate the link between symptom-tracking apps, behavior-tracking apps, contact tracing, aggregate population mobility monitoring, healthcare access and e-health monitoring^40^.

Nevertheless, this kind of monitoring raises important ethical issues, as it could be considered as limiting individual freedoms and rights^43–46^. In our case, monitoring of these patients with wearable devices was already established as part of the ongoing MEDEA project that started much before the spread of COVID-19, and thus we had obtained timely ethical approval and written consent from participants. In the case of pandemics, where decisions and measures are taken within days or even hours, it is extremely difficult to obtain fast-track consent from individuals to record their attitudes with wearable technology. For this reason, future health surveillance tracking and contact-tracing tools and applications should be designed in a way that can preserve individual freedoms and assert patient autonomy in a socially responsible manner, while educating the population about the positive effects of health surveillance^47^.

An important limitation of wearable technology is the loss of signal of GPS tracking, especially in indoor environments, which introduces the challenge of how to treat missing values. As a response, automated microenvironment classification algorithms that include spatial and temporal buffering have been developed and validated, especially for air pollution exposure studies^48^ and provide an effective way to account for missing location data. Our findings should be interpreted with caution and not be generalized directly to all children or to the general population, because soon after the appearance of the COVID-19 pandemic it became widely known that asthmatic patients may be at increased risk for severe disease^49^. Furthermore, asthmatic patients were more likely accustomed to protective behavioural changes even before the pandemic, as they were aware of the negative health effects of poor air quality^50,51^ and the potential of atmospheric pollutants to further impair the respiratory system and facilitate viral infection, as it has been suggested for SARS-CoV-2^52^. These factors may have led to increased compliance to public health intervention measures for COVID-19, as compared to the general population. Further studies, focusing on the general population are needed to better assess the full extent of population compliance to public health intervention measures for COVID-19. Finally, our cohort lacks information about family characteristics during the pandemic, such as parents’ employment status and time spent working from home, which may affect the outcomes of our study.

## Conclusion

In conclusion, we implemented novel wearable technology methods to assess personal compliance to public health interventions aiming to contain the spread of a novel, highly contagious virus such as SARS-CoV-2. The successful implementation of public health interventions in Cyprus and Greece, which minimized COVID-19 related mortality in both countries, was reflected in the sharp mobility reductions recorded in the participants of our study early in the course of the outbreak. Wearable devices provide objective, continuous, real-time data that may timely inform public health officials on compliance to various tiers of public health interventions and ensure informed decision-making and strategic planning in the containment of epidemics, both at national and cross-national levels.

## Materials and methods

### Study setting

Asthmatic children were recruited from primary schools in Cyprus and Greece (Heraklion district, Crete) and were enrolled in the ongoing LIFE-MEDEA public health intervention project (Clinical.Trials.gov Identifier: NCT03503812). The LIFE-MEDEA project aims to evaluate the efficacy of behavioral recommendations to reduce exposure to particulate matter during desert dust storm (DDS) events and thus mitigate disease-specific adverse health effects in vulnerable groups of patients. Details of the study protocol and methods are presented in Supplementary File 1. In order to assess adherence to recommendations, participants are equipped with a wearable device (smartwatch) with several sensors. Participants are instructed to wear the smartwatch throughout the study period, during both DDS and non-DDS days. During non-DDS days, all participants carry out their usual daily activities. The first MEDEA study period took place during February-June 2019, the second study period during February-June 2020.

### Study populations and recruitment

In the asthma panel study the target population were children aged from 6 to 11 years with mild to moderate persistent asthma. The eligibility criteria included a physician’s diagnosis of asthma and at least one of the following: daily preventative asthma medication, wheezing episodes and/or unscheduled medical visits for asthma during the past 12 months. Basic demographic and clinical information from all participants was collected during the recruitment process. The number of eligibility criteria applying for each participant were used to categorize participants into asthma severity groups. The groups were defined as follows: Asthma Severity 1 (Physician diagnosis plus one other eligibility criterion), Asthma Severity 2 (Physician diagnosis plus two other eligibility criteria) and Asthma Severity 3 (Physician diagnosis plus three or more other eligibility criteria). In Cyprus, study approvals were obtained from the Cyprus National Bioethics Committee (EEBK EΠ 2017.01.141), the Data Protection Commissioner (No. 3.28.223) and the Ministry of Education (No 7.15.01.23.5). In Greece, approvals were obtained from the Scientific Committee (25/04/2018, No: 1748) and the Governing Board of the University General Hospital of Heraklion (25/22/08/2018). Guardians of all participants provided written informed consent and all methods described below were performed in accordance with the relevant guidelines and regulations.

### Physical activity and GPS tracking

Physical activity and global positioning system (GPS) data were recorded between February 3 and April 26, for both study years (2019 and 2020) in Cyprus and Greece using the smartwatch. Data from study year 2020 were extracted to assess the participants’ mobility before and during the enforcement of COVID-19 lockdown measures while data from study year 2019 that represent a completely restrictions-free mobility period were also used for comparison. The EMBRACE™ smartwatch (Embrace Tech LTD, Cyprus) was used for data collection. The smartwatch works as a stand-alone device and is equipped with multiple sensors such as pedometer, GPS and heart rate as well as an embedded sim-card for Wi-Fi data transfer. The software is capable of synchronizing the sensors, so the data are transferred to the cloud with the same timestamp. Data on GPS coordinates and steps/time unit, and heart rate are collected per 5-min intervals. Data synchronization with a cloud-based database is performed automatically when the smartwatch contacts the Wi-Fi network inside the participants’ home. For each participant we recorded the total number of steps per day (24-h period). In addition, we defined the fraction of time spent at home as the ratio of time with GPS signal within a 100 m radius around the participant’s residence divided by 24 h. The 100 m radius was defined as the maximum barrier to account for the accuracy of GPS signal in commercially available GPS receivers^53^. As signal accuracy in urban and especially indoor environments is further blocked or bounced repeatedly off buildings prior to being received^54^, we also classified 5-min intervals with no GPS signal as either “at-home” or “out-home”, depending on the signal of the most recent valid GPS recording. Lastly, the days that participants did not wear the smartwatch were identified by absence of heart rate measurements, and were excluded from subsequent analysis. We also excluded from the analysis GPS and pedometer data for DDS days in Cyprus (5 days during February-April 2020 and 4 days during February-April 2019) and Crete-Greece (1 day during February-April 2020 and 2 days during February-April 2019) that may had further influenced the mobility of the participants.

### Public health (non-pharmaceutical) interventions in Cyprus and Greece

**Figure 3:**
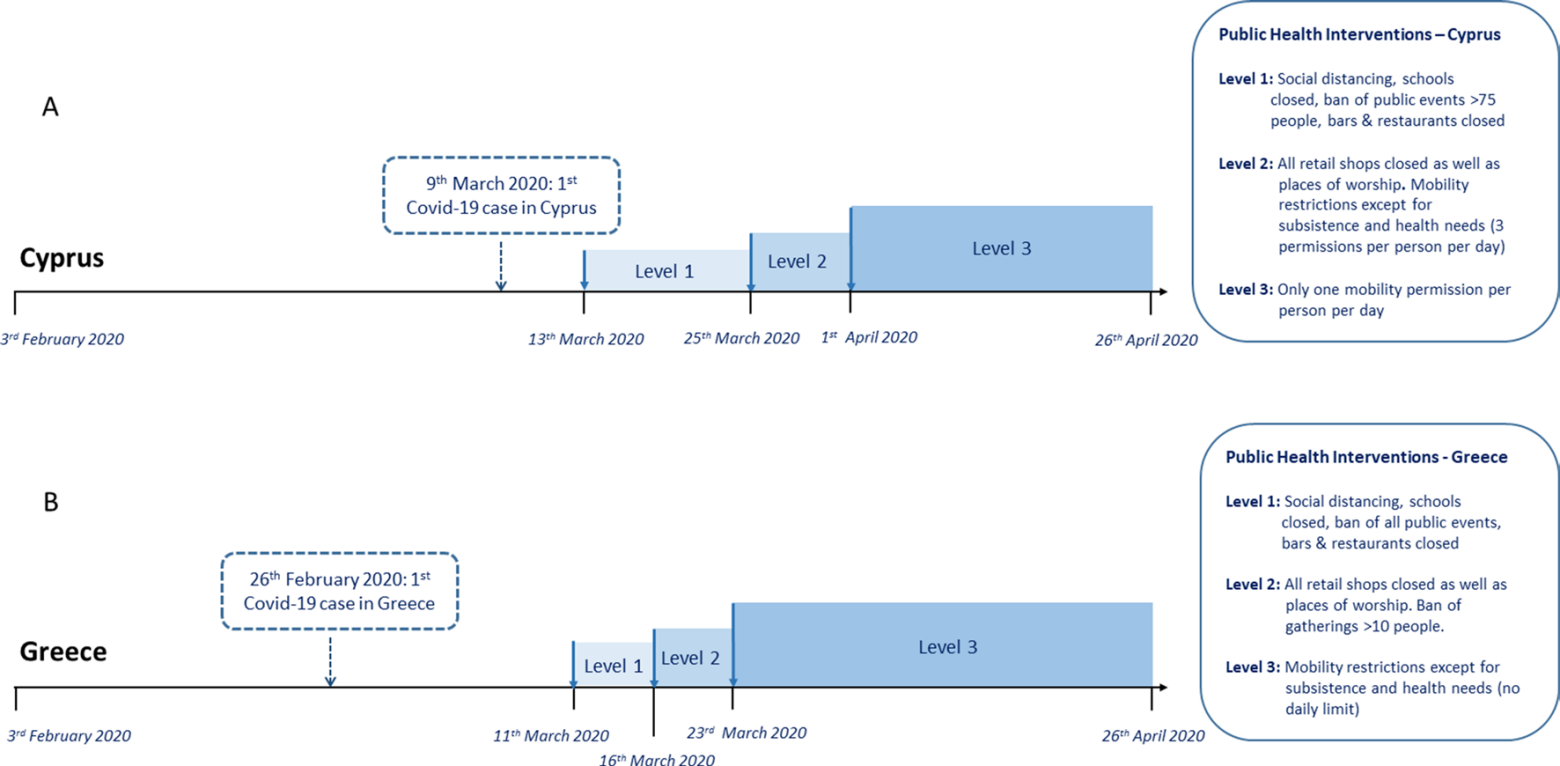
Timeline of public health interventions in Cyprus and Greece. Timeline of the study recordings in relation to introduction of public health interventions in Cyprus (**A**) and Greece (**B**) during March–April 2020.

Data collection period spans for 12 weeks from February 3 to April 26, 2020 and was divided into four levels based on the implemented public health interventions in each country. In Cyprus, the first COVID-19 cases were identified on March 9, 2020 and the study period is divided to: i) level 0 (baseline)—no public health interventions (Weeks 1–5: February 3, 2020–March 12, 2020), ii) level 1—social distancing measures, ban of public events with > 75 people, bars, restaurants and schools closed (Weeks 6–7: March 13, 2020–March 24, 2020), iii) level 2—all retail shops and worship places were closed, and mobility restrictions were implemented, except for subsistence and health needs (3 permissions per person per day) (Week 8: March 25, 2020–March 31, 2020), iv) level 3—stringent lockdown with only one mobility permission per person per day (Weeks 9–12: April 01, 2020–April 26, 2020) (Fig. 3). In Greece, the first COVID-19 case was identified on February 26, 2020 and the study period is divided to: i) level 0 (baseline)— no public health interventions (Weeks 1–5: February 03, 2020–March 10, 2020), ii) level 1—social distancing measures, ban of all public events, bars, restaurants and schools closed (Week 6: March 11, 2020–March 15, 2020), iii) level 2—all retail shops and worship places closed, ban of gatherings of > 10 people (Week 7: March 16, 2020–March 22, 2020), iv) level 3—mobility restrictions except for subsistence and health needs (no daily limit) (Weeks 8–12: March 23, 2020-April 26, 2020) (Fig. 3).

### Statistical analysis

Basic demographics and clinical characteristics of the asthmatic children were summarized using mean (standard deviation) for continuous variables and percentages for categorical variables. In an unadjusted analysis, the mean fraction time spent at home and mean total steps per day were compared between the different periods of public health interventions using ANOVA, while graphs were constructed in order to demonstrate the weekly variation in mobility before and during the implementation of public health interventions in Cyprus and Greece. Furthermore, the course of fraction time spent at home and total steps per day in asthmatic children participating in the second MEDEA study period during February–April 2020 and the same parameters’ course in asthmatic children participating in the first MEDEA study period during February–April 2019 are displayed in separate graphs for Cyprus and Greece.

The changes in the daily levels of fraction time spent at home and total steps were further explored in a mixed effect model, which included a fixed effect term for the level of public health interventions and a random intercept for each participant. The mixed effect model was adjusted for the effect of age, gender, temperature, humidity, year, and weekend on mobility. In addition, sine and cosine functions were included to control for monthly variability in our data. Finally, for each parameter, we used an interaction term to test for differential change of mobility across the levels of interventions measures.

All statistical comparisons were performed using STATA 12 (StataCorp, TX) and a *p* value lower than 0.05 was considered as statistically significant.

## Supplementary Information

Supplementary Information

## Data Availability

A preprint of the manuscript is available under https://www.researchsquare.com/article/rs-37518/v1. The original data are available under https://zenodo.org/record/3906445#.XvUBhG5uI2w (https://doi.org/10.5281/zenodo.3906445).
