# Supplementary material for: Use of wearable sensors to assess compliance of asthmatic children in response to lockdown measures for the COVID-19 epidemic
Source: Sci Rep. 2021 Mar 15;11:5895. doi: 10.1038/s41598-021-85358-4 (PMC7971022; doi:10.1038/s41598-021-85358-4)
Supplement: Supplementary file 1 — Supplementary Information [file 41598_2021_85358_MOESM1_ESM.docx]

**Supplementary Information**

**Use of wearable sensors to assess compliance of asthmatic children in response to lockdown measures for the COVID-19 epidemic**

Panayiotis Kouis, Antonis Michanikou, Pinelopi Anagnostopoulou, Emmanouil Galanakis, Eleni Michaelidou, Helen Dimitriou, Andreas Matthaiou, Paraskevi Kinni, Souzana Achilleos, Harris Zacharatos, Stefania I. Papatheodorou, Petros Koutrakis, Georgios K. Nikolopoulos, Panayiotis K. Yiallouros

**Study approvals from Ethics and National authorities**

The MEDEA Asthma panel studies have been registered with and approved by the clinicaltrials.gov online repository (ClinicalTrials.gov Identifier: NCT03503812) and relevant authorities at both Cyprus and Greece, according to national legislation. In Cyprus, approvals have been obtained from the Cyprus National Bioethics Committee (EEBK EΠ 2017.01.141), by the Data Protection Commissioner (No. 3.28.223) and Ministry of Education (No 7.15.01.23.5). In Greece, approvals have been obtained from the Scientific Committee (25/04/2018, No: 1748) and the Governing Board of the University General Hospital of Heraklion (25/22/08/2018).

**Details on LIFE-MEDEA study protocol and methods**

***Recruitment protocol - Recruiting schools***

During November-December of 2018 and 2019, schools in Cyprus (Nicosia, Limassol) and Greece (Heraklion), were engaged with the LIFE-MEDEA project in collaborative effort to promote health education within the school and community setting. For this purpose, interactive and age-appropriate presentations were given to schoolchildren, focusing on healthy eating habits, respiratory health and the impact of climate change on human health. Following the presentations, the International Study of Asthma and Allergies in Children (ISAAC) questionnaire, enriched with questions on medical care and medication utilization, was sent to the parents through their children to be filled and in turn, the filled questionnaire was sent back to the school administration and LIFE-MEDEA project through the schoolchildren. Based on the replies to the questionnaire, we detected eligible asthmatic children for participation in the study during the high DDS periods of February-May 2019 and 2020. Parents of eligible schoolchildren were then informed by LIFE-MEDEA personnel about the study and were asked to participate.

**Parallel intervention groups**

Participants were randomized to three parallel intervention groups to receive: a) no additional intervention for DDS (control group), b) LIFE-MEDEA project interventions for outdoor exposure reduction, and c) LIFE-MEDEA project interventions for both outdoor and indoor exposure reduction. In summary, in outdoor intervention, participants were asked to stay indoors, as well as to avoid intense physical activity outdoors, competitive sports and unnecessary walks during DDS days, while in indoor intervention participants were also asked to close windows and doors, seal possible cracks around windows and doors in order to minimize home ventilation, and use continuously an air cleaner in order to filter indoor air. In both intervention groups, patients were alert about upcoming DDS days by early dissemination of warnings and audiovisual instructions through the bidirectional, patient-centered web-based platform of LIFE-MEDEA (MEDena® Health-Hub). Participants in the control group received no warnings about upcoming DDS days.

***Baseline and follow-up clinical assessments***

Eligible asthmatic children and their parents were invited for a baseline clinical assessment in January 2019 and January 2020 prior to the onset of the respective high DDS periods. During the baseline visit, we collected additional sociodemographic and clinical data about the patients as well as additional information about classroom and home environmental characteristics, including information about second-hand tobacco smoke exposure.

The follow-up period, spanning from February to late May of 2019 and 2020, included continuous monitoring of the participants’ daily location and physical activity using the wearable sensors. Phone interviews were conducted at baseline and then at every 1-month interval throughout the high DDS period. Interviews were performed in order to collect information on asthma symptoms control, medication use and unscheduled visits to health professionals for asthma. Asthma symptoms control in the past month was assessed using the English (if required) and Greek versions of the pediatric Asthma Control Test (c-ACT, license number: QM044906) as used previously [1-3]. In addition, according to the protocol, participants for the year 2019 underwent lung function assessment (In2itive Spirometer, Vitalograph Ltd, United Kingdom) and Fractional Exhaled Nitric Oxide (FeNO - NIOX VERO portable nitric oxide analyzer, Circassia, United Kingdom), at baseline, mid-period (April) as well as at the end of the follow up period (late May). Lastly, at the end of the follow up period skin prick testing to 14 common aero-allergens (Allergy Therapeutics PLC, United Kingdom) was performed as described previously [4]. Participants for the year 2020 underwent only the baseline lung function test, considering that the other two time-points coincided with the Covid-19 pandemic, during which lung function testing was prohibited, according to international guidelines.

***Asthma morbidity outcomes and data analysis***

Within the framework of LIFE-MEDEA project, the ACT questionnaire score is considered as the primary health outcome and a change of three points in the total score is considered clinically meaningful [2,5]. For the primary analysis, the combined effect in the two intervention groups will be compared versus the control group and next, we will compare the effect between each of the intervention groups and the control group as well as between each intervention group. Secondary health outcomes will be the presence or absence of asthma symptoms in the previous month, asthma medication use, unscheduled visits for asthma, and values of forced expiratory volume in 1 second, peak expiratory flow and FeNO.

***Sample size calculations for asthma panel study***

The childhood ACT is comprised of seven items and a score ranging from 0 (poorest asthma control) to 27 (optimal asthma control) can be calculated with a cut-off point of 19 indicating uncontrolled asthma [5]. Previous studies have shown that a minimally meaningful change in the ACT score is three points [2]. To detect a statistically significant difference of three points, and assuming a 30% dropout rate, the minimum sample size required in each of the three parallel groups was 100 participants. This sample size calculation is based on a level of 0.05 and a power of at least 80% to detect this difference between the comparison groups.

**Supplementary Table 1**: Observed fraction time spent at home and total steps per day across levels of intervention for Covid-19 in Cyprus and Greece for all participants and for each asthma severity category separately. Values are presented as mean (95% Confidence Interval)

|  | | **Fraction time spent at home** | | | | **Steps per day** | | | |
| --- | --- | --- | --- | --- | --- | --- | --- | --- | --- |
| **Intervention** | | **All children**  **(n=53)** | **Asthma severity 1**  **(n=25)** | **Asthma severity 2**  **(n=20)** | **Asthma severity 3**  **(n=8)** | **All children**  **(n=53)** | **Asthma severity 1**  **(n=25)** | **Asthma severity 2**  **(n=20)** | **Asthma severity 3**  **(n=8)** |
| **Asthmatic children (Cyprus)**  **(n=53)** | **Baseline**  **(Level 0)** | 43.8%  (40.5%; 47.1%) | 39.4%  (34.1%; 44.7%) | 42.9%  (37.9%; 48.1%) | 43.9%  (33.1%; 54.7%) | 8996  (8567; 9425) | 8879  (8144; 9613) | 8388  (7788; 8988) | 9895  (8639; 11151) |
|  | **Level 1** | 88.9%  (85.7%; 92.1%) | 90.5%  (86.4%; 94.5%) | 87.1%  (80.1%;94.2%) | 85.9%  (74.7%; 97.1%) | 6499  (5832; 7166) | 6520  (5639; 7483) | 5129  (4492; 5766) | 9387  (7369; 11404) |
|  | **Level 2** | 95.5%  (93.8%; 97.2%) | 96.5%  (95.4%; 97.6%) | 95.5%  (92.1%;99.0%) | 96.6%  (91.2%; 99.9%) | 6248  (5683; 6812) | 6389  (5490; 7288) | 4965  (4275; 5656) | 7995  (5999; 9990) |
|  | **Level 3** | 94.1%  (92.5%; 95.7%) | 95.5%  (93.3%; 97.7%) | 96.2%  (94.9%;97.5%) | 74.6%  (55.5%;93.7%) | 6270  (5814; 6727) | 5503  (4763; 6243) | 5471  (5006; 5936) | 8111  (5238; 10985) |
|  | | **Fraction time spent at home** | | | | **Steps per day** | | | |
| **Intervention** | | **All children**  **(n=55)** | **Asthma severity 1**  **(n=21)** | **Asthma severity 2**  **(n=26)** | **Asthma severity 3**  **(n=8)** | **All children**  **(n=55)** | **Asthma severity 1**  **(n=21)** | **Asthma severity 2**  **(n=26)** | **Asthma severity 3**  **(n=8)** |
| **Asthmatic children (Greece)**  **(n=55)** | **Baseline**  **(Level 0)** | 52.4%  (49.4%; 55.4%) | 56.5%  (52.4%; 60.7%) | 49.1%  (43.4%; 54.7%) | 49.2%  (42.6%; 55.7%) | 8527  (8145; 8908) | 9178  (8632; 9725) | 7843  (7090; 8595) | 7686  (6950; 8421) |
|  | **Level 1** | 71.4%  (60.4%; 82.5%) | 69.7%  (52.1%; 87.3%) | 79.8%  (59.1%; 99.0%) | 66.2%  (56.6%; 75.8%) | 6864  (5689; 8040) | 6935  (5139; 8731) | 5332  (3649; 7015) | 5899  (3603; 8194) |
|  | **Level 2** | 84.9%  (80.3%; 89.4%) | 84.6%  (78.0%; 91.1%) | 90.3%  (83.9%; 96.6%) | 71.0%  (65.8%; 76.2%) | 5533  (4769; 6297) | 5792  (4562; 7023) | 5194  (3807; 6580) | 4652  (3446; 5858) |
|  | **Level 3** | 89.6%  (87.0%; 92.3%) | 89.4%  (85.5%; 93.3%) | 88.0%  (81.4%; 94.7%) | 91.9%  (88.3%; 95.5%) | 5439  (5051; 5829) | 5763  (5202; 6323) | 4868  (4048; 5688) | 5460  (4668; 6251) |

**References**

1. Grammatopoulou EP, Skordilis EK, Stavrou N, Myrianthefs P, Karteroliotis K, Baltopoulos G, Koutsouki D: **The effect of physiotherapy-based breathing retraining on asthma control.**Journal of Asthma 2011, **48**(6):593-601.
2. Voorend-van Bergen S, Vaessen-Verberne AA, Landstra AM, Brackel HJ, van den Berg, Norbert J, Caudri D, de Jongste JC, Merkus PJ, Pijnenburg MW: **Monitoring childhood asthma: web-based diaries and the asthma control test.**J Allergy Clin Immunol 2014, **133**(6):1599-1605. e2.
3. Koumpagioti D, Boutopoulou B, Priftis KN, Douros K: **Effectiveness of an educational program for children and their families on asthma control treatment adherence.**Journal of Asthma 2020, **57**(5):567-573.
4. Kolokotroni O, Middleton N, Gavatha M, Lamnisos D, Priftis KN, Yiallouros PK: **Asthma and atopy in children born by caesarean section: effect modification by family history of allergies–a population based cross-sectional study.**BMC pediatrics 2012, **12**(1):179.
5. Liu AH, Zeiger R, Sorkness C, Mahr T, Ostrom N, Burgess S, Rosenzweig JC, Manjunath R: **Development and cross-sectional validation of the Childhood Asthma Control Test.**J Allergy Clin Immunol 2007, **119**(4):817-825.
